# Supplementary material for: A single clonal lineage of transmissible cancer identified in two marine mussel species in South America and Europe
Source: eLife. 2019 Nov 5;8:e47788. doi: 10.7554/eLife.47788 (PMC6831032; doi:10.7554/eLife.47788)
Supplement: Supplementary file 1. [file elife-47788-supp1.docx]

Supplementary File 1. Table of PCR primers used for amplification and sequencing

| Locus | Forward primer | Reverse primer | Annealing (°C) | Size (bp) |
| --- | --- | --- | --- | --- |
| *EF1α* | consEF1-F1  ACCATTGATATTGCTYTNTGGAA | MtEF1R1 (used for some *M. chilensis*)  TGTTCTCTGGTCTGACCGTT  MtEF1-R8  CCGTTGGATGAGATNCCNGCYTC | 50 | ~470-618 |
| *H4* | 5-H4-Full  ATTCCTACAGAGTTACCTCCCGGAT | 3-H4-Full  AAGTTGGACAAGTTGGACAGGAGA | 52 | ~550-556 |
| mtCR | AB15  TTGCGACCTCGATGTTGG | AB1639  CAGGCTRTARAGCATAATCTAAAAC  AB1639B (used for some *M. chilensis*)  CAGGCTRTARAGCATAATCTAAAACRAG | 50 | ~702-732 |
| mt*COI* | LCO1490MspD2  GRTCAACAAATCAYAAAGATATTGG | HCO-flankMspR3  CTGCTTCCTTTCCGGAGCAATG | 50 | ~733 |
