# Supplementary material for: A single clonal lineage of transmissible cancer identified in two marine mussel species in South America and Europe
Source: eLife. 2019 Nov 5;8:e47788. doi: 10.7554/eLife.47788 (PMC6831032; doi:10.7554/eLife.47788)
Supplement: Supplementary file 2. [file elife-47788-supp2.docx]

Supplementary File 2. Table of qPCR primers used

| Target | Forward primer | Reverse primer | Amplicon length |
| --- | --- | --- | --- |
| *EF1α*  (control) | MspEF1qF3B  TGGAAGTTTGAGACCACCAAATACT | MspEF1qR3B  TTACACTCACCAGTGATCATGTTCTT | 92 bp |
| *EF1α*  (H allele-specific) | Mch-MF2(130)B  GCAAAAGTGGCTGAAAACCAGATTCTA | MchC-HR2C  GTAAAAAAGTTAAAATTTCTTTTAGTCACACAAT | 180 bp |
| *H4*  (control) | Msp-H4-qF1b  CCAGCAATCCGTCGTTTAGC | Msp-H4-qR1  CAGGGCGTAGACAACATCCA | 176 bp |
| *H4*  (MR allele-specific) | Mch-H4-MRF3  CAATCCGTCGTTTAGCAAGAC | Mch-H4-MRR1  ACAGGAGAAAGCTATGGTTTTCT | 322 bp |
| *H4*  (KNS allele-specific) | Mch-H4- SKF6  TTCGTTTTATCGAACTTCAC | Mch-H4-SKR3  TACGTTTGACACCACCTCG | 170 bp |
| *mtAB*  (control) | MspmtAB-F1  GGTCTGTTCGCCCTTTAAAATCT | MspmtAB-R1  TGAACTAGCTCACGCCGTTC | 59 bp |
| *mtAB*  (C allele-specific) | MchmtAB-CF1  ACCCTTAAGAATGAGGTTTTAC | MchmtAB-CR1  CATGCATCAGCAACAGGGTTTTG | 113 bp |
| *mtAB*  (D allele-specific) | MchmtAB-DF6  GTACTTCATTTCCTTGCCA | MchmtAB-DR2  AAAAGACAGGTGGAAAGGGGT | 60 bp |
| mt*COI*  (control) | COI-Msp-qF2  AGGATAGATGTTCTTATTRTGTC | COI-Msp-qR2d  CATAAAGCTCMGCTCGTTCTCCT | 130 bp |
| mt*COI*  (B allele-specific) | COI-M-B-qF1  AATACTGTCCTTTAGAACAGAC | COI-M-B-qR1b  CTAGGACCCCTATGATGG | 89 bp |
